# A Trifluoromethyl Quinazoline Compound Regulates the Epithelial–Mesenchymal Transition of Prostatic Hyperplasia Cells by Inhibiting the Secretion of TGF-β1 in Stromal Cells

**DOI:** 10.3390/cimb47121057

**Published:** 2025-12-17

**Authors:** Lu Chen, Di Zhang, Gang Yu, Sha Cheng, Bixue Xu, Jia Yu, Jiming Liu, Heng Luo

**Affiliations:** 1 State Key Laboratory of Discovery and Utilization of Functional Components in Traditional Chinese Medicine, Guizhou Medical University, Guiyang 550014, China; chenlu546666@163.com (L.C.); badadi003@163.com (D.Z.); yugang@gzcnp.cn (G.Y.); chengsha@gzcnp.cn (S.C.); bixue_xu@126.com (B.X.); yujia@gzcnp.cn (J.Y.); 2 Natural Products Research Center of Guizhou Province, Guiyang 550014, China

**Keywords:** benign prostatic hyperplasia (BPH), epithelial–mesenchymal transition (EMT), quinazoline derivatives, TGF-β1, β-tubulin

## Abstract

Benign prostatic hyperplasia (BPH) is a common disease in elderly men; its occurrence is closely related to the interaction between stromal cells and epithelial cells in the prostate. This article aims to explore the potential therapeutic effect and mechanism of a new trifluoromethyl quazoline compound (kzl054) on BPH. The results showed that kzl054 had inhibitory activity that limited the growth of prostate hyperplasia cells, BPH-1, and stromal cells, WPMY-1. It could also induce apoptosis of BPH-1 cells and arrest their cell cycle. animal experiment results showed that kzl054 could effectively reduce the volume and prostate index of mouse prostate hyperplasia tissues. Through the establishment of a co-culture system of BPH-1 and WPMY-1 cells, it was found that co-culture could induce EMT in BPH-1 cells. While kzl054 could affect the secretion of TGF-β1 by competitively binding to the colchicine binding site on β-tubulin and inhibiting the expression of β-tubulin, through inhibiting the secretion of TGF-β1 by stromal cells. This study has revealed that compound kzl054 inhibits the secretion of TGF-β1 by targeting the inhibition of microtubule polymerization and regulating the epithelial cell EMT, providing potential candidate molecules and mechanisms for the development of new drugs for the treatment of BPH.

## 1. Introduction

Benign prostatic hyperplasia (BPH) is a common chronic disease among elderly men. It is characterized by abnormal benign growth of the prostate, often leading to urinary system symptoms such as frequent urination, urgent urination, and difficulty in urination, and can also cause complications such as urinary tract infection, urinary calculi, and urinary incontinence [1,2]. These symptoms seriously affect the quality of life of patients. The mechanism of BPH is not yet fully understood, but it is generally believed to be related to sex hormones such as testosterone and growth factors associated with aging [3,4]. The main purpose of BPH treatment is to reduce the volume of the prostate and improve lower urinary tract symptoms. Non-pharmacological treatments include lifestyle changes, surgical excision or minimally invasive surgery, but these treatments can lead to adverse reactions such as erectile dysfunction, bleeding and urinary incontinence [5,6]. In addition, minimally invasive surgery is only suitable for smaller prostates and usually has a relatively high recurrence rate [7]. Drug therapy typically includes alpha-receptor blockers and 5-α-reductase inhibitors, which are clinically effective but often come with side effects, including an increased risk of sexual dysfunction, prostate cancer, heart failure and depression. Approximately 30% of patients experience disease progression or drug resistance [8,9,10,11]. In addition, modern lifestyle factors, diet and environmental pollution have led to a younger age of onset for BPH patients, and adverse reactions such as sexual dysfunction and depression may affect the use of these drugs [12]. Therefore, exploring new strategies for treating BPH and minimizing drug-related side effects is a key issue in current BPH management.

Studies have shown that BPH is the result of continuous proliferation and inhibition of apoptosis of prostate stromal and epithelial cells, leading to an imbalance between proliferation and apoptosis. During this process, growth factors such as fibroblast growth factor (FGFs), insulin-like growth factor (IGF), and transforming growth factor β (TGF-β1) play a crucial regulatory role [13,14,15]. It is notable that epithelial-mesenchymal transition (EMT) induced by growth factors such as TGF-β1 plays a key role in the excessive proliferation of prostate epithelial cells [16,17]. EMT controls the differentiation process of the normal urogenital system. During the development of the prostate, stromal cells regulate the proliferation and differentiation of epithelial cells through paracrine signaling [18]. Therefore, targeting the interaction between the matrix and epithelial cells as well as EMT transformation is of great significance for improving BPH.

Quinazoline derivatives and microtubule inhibitors have indeed been extensively explored in the research of prostate diseases. Quinazoline alkaloids are a class of benzopyridine heterocyclic compounds widely present in nature, possessing a wide range of biological activities such as anti-tumor, anti-inflammatory, antibacterial, anti-tuberculosis, and anti-diabetic [19]. The quinazoline core, as an advantageous structural framework for drug development, is widely used in the research of anti-tumor drugs, among which the research on epidermal growth factor receptor (EGFR) tyrosine kinase inhibitors (TKIs) is the most active [20]. Therefore, how to develop new drugs with unique chemical structures and new mechanisms of action based on this advantageous structural framework of quinazoline has significant theoretical research significance and application value. Studies have shown that due to the fact that 2-position unsubstituted quinazoline compounds may be oxidized and metabolized by aldehyde oxidase in vivo [21]. The research group combined the characteristics of fluorine atoms and their wide application in drugs: fluorine has the strongest electromagnetically (4.0) and a generally similar atomic radius to hydrogen atoms (1.47 Å), as well as a very strong C–F bond (averaging approximately 116 kcal/mol), etc. These chemical properties make the substitution of other atoms in a compound by a fluorine atom often cause significant changes in its physical, chemical, and biological activity properties. This enables fluorine-containing compounds to be widely used in many fields, especially in the pharmaceutical field, and in recent years, fluorine-containing drug molecules account for approximately 20% to 30% of the newly approved drugs [22]. Trifluoromethyl (-CF3) is a common fluorine-containing group; introducing it into the compound molecule can significantly modify the chemical stability, lipophilicity and hydrophilicity, biolipid permeability, in vivo metabolic stability, and its binding ability to biological proteases of the compound [23].

During the process of cell proliferation, microtubules form a spindle during mitosis, promoting chromosome separation to form two daughter cells [24]. Microtubules are composed of α-tubulin and β-tubulin, which are arranged end to end to form hollow tubes and are self-assembled proteins within cells. Microtubule inhibitors, including paclitaxel and colchicine, are commonly used as chemotherapy drugs by interfering with microtubule dynamics, inducing apoptosis and blocking mitosis [25,26]. In addition to their role as chemotherapy drugs, microtubule inhibitors have been reported to be closely related to inflammatory and fibrotic processes [27]. For instance, colchicine is still widely used in the treatment of inflammation and tumors and has been proven to interact with factors such as TGF-β1 [28,29]. The research on quinazoline-based microtubule inhibitors in the field of oncology has been extensive. The novelty of our study does not lie in discovering a completely new drug category, but rather in innovatively applying a well-validated target (the microtubule system) and its chemical probes (a new quinazoline derivative, kzl054, which introduces a trifluoromethyl functional group at different sites of the quinazoline parent nucleus) to address a key pathological aspect that is not adequately addressed by existing therapies in benign prostatic hyperplasia—namely, interstitial fibrosis and tissue remodeling. While microtubule-targeting agents are well-established in oncology, their application in benign hyperplastic conditions like BPH remains underexplored. Given the central role of cytoskeletal remodeling in stromal activation and tissue fibrosis—key pathological features of BPH—we hypothesized that a novel quinazoline derivative, kzl054, might exert therapeutic effects by modulating β-tubulin dynamics. This study aims to evaluate the efficacy of kzl054 in a preclinical BPH model and to investigate its potential mechanism of action, focusing on the interplay between β-tubulin stabilization, TGF-β1 signaling, and epithelial-stromal crosstalk, thereby exploring a potential new therapeutic strategy that addresses the structural remodeling component of BPH. This study explores the microtubule inhibitor kzl054 targeting β-tubulin polymerization, down-regulating the expression and secretion of TGF-β1 in WPMY-1 cells, and inhibiting EMT transformation in BPH-1 cells, thereby regulating the proliferation and progression of BPH cells in vivo and providing new insights for BPH treatment.

## 2. Materials and Methods

### 2.1. Compounds and Cell Culture

The trifluoromethyl quinazoline derivative kzl054 [30] is derived from the Natural Products Research Center of Guizhou Province. BPH-1 and WPMY-1 cells were stored in the Biology laboratory of the Natural Products Research Center of Guizhou Province (Guiyang, China). All cells were maintained in RPMI-1640 (Hyclone, Logan, UT, USA) supplemented with 10% fetal bovine serum (Sijiqing, Hangzhou, China) and 1% penicillin and streptomycin (Solarbio, Beijing, China). All cell lines utilized in this study were procured from reputable cell banks (e.g., ATCC, DSMZ, or the Cell Bank of the Chinese Academy of Sciences). To ensure cell line authenticity, short tandem repeat (STR) profiling was performed on all cell lines both prior to experimentation initiation and within three months following its completion. The STR profiles demonstrated exact genotype matches (match score > 85%) against reference profiles in the respective databases. Furthermore, cell lines employed for transfection were routinely screened using a commercial mycoplasma detection kit and verified to be Mycoplasma-free. Corresponding STR profile data for the specific cell lines are available upon request.” Additionally, the rationale for selecting the specific concentrations used is based on systematic dose–response preliminary experiments. Prior to definitive assays, cells were treated with an extended concentration gradient (e.g., 0.1 nM, 1 nM, 10 nM, 100 nM, 1 µM, 2 µM, 4 µM, 8 µM) of the compound, and key effect markers were assessed (e.g., cell viability, target protein expression levels, or specific functional readouts). These preliminary data revealed a significant dose-dependent response within the 0.1 nM–8 µM range. Specifically, treatment with 1 µM induced approximately 50% of the maximal effect in WPMY-1 cells, while exhibiting acceptable cytotoxicity (viability > 85%). Consequently, 1 µM was selected as the screening concentration for initial compound activity assessment, followed by detailed characterization of the dose-activity relationship within the 0.1 nM–2 µM range to calculate the IC_50_ and evaluate the concentration dependence of cell growth inhibition. Separately, for the animal studies, prior to definitive experimental cohorts, small-scale dose-finding experiments were conducted (administering 10, 25, 50 mg/kg). Based on these findings, doses of 30 mg/kg and 60 mg/kg administered for 4 weeks elicited significant pharmacodynamic biomarker changes indicative of efficacy without inducing observable body weight loss or behavioral abnormalities. These selected doses were subsequently employed in the definitive animal experiments to evaluate the compound’s effect on prostate hyperplasia in vivo.

### 2.2. MTT Assay for Cell Proliferation

BPH-1 and WPMY-1 cells were seeded in 96-well plates. kzl054 was dissolved in DMSO, and the compound was added at different concentrations for 48 h and 72 h. After treatment, MTT solution (Solarbio, China) was added to each well for 4 h. Absorbance was measured at 490 nm using a microplate reader (Gene, Hong Kong), and the inhibition rate was calculated by the formula: (1 − experimental group OD/control group OD) × 100%.

### 2.3. Flow Cytometry for Cell Cycle and Apoptosis Detection

All cells were seeded in 60 mm culture dishes and treated with RMPI-1640 medium containing different compounds (the control group received 1% DMSO). After 48 h, cells were digested with trypsin and collected. Subsequently, 5 μL propidium iodide (Solarbio, China), 10 μL FITC (Solarbio, China), and RNAse were added, and the mixture was incubated in the dark at room temperature for 30 min. After filtering through a cell strainer, flow cytometry (Agilent, Santa Clara, CA, USA) was performed to detect the cell cycle and apoptosis.

### 2.4. Colony Formation Assay

BPH-1 and WPMY-1 cells were seeded in 6-well plates. Different active compounds were added to treat the cells for 48 or 72 h, until the control group had more than 50 colonies. After fixing with fixative solution (Solarbio, China), crystal violet staining (Solarbio, China) was performed, and colonies were counted after drying.

### 2.5. Establishment of Prostatic Hyperplasia Model

Rats and mice were allowed to recover for 7 days after castration. Starting from day 8, a daily subcutaneous injection of a mixed oil formulation (sesame oil) containing testosterone propionate (5 mg/kg) and estradiol benzoate (50 µg/kg) was administered for 4 consecutive weeks to induce benign prostatic hyperplasia. This dosage regimen, referenced from classical literature [31,32], was validated in our laboratory’s preliminary experiments to consistently induce models with histopathological features consistent with benign prostatic hyperplasia, demonstrating good animal tolerance.

### 2.6. Animal Experiments

Male C57BL/6J mice were purchased from Zhejiang Weitong Lihua Experimental Animal Technology Co., Ltd. (Hangzhou, China; License No. SCXK (Zhe) 2019-0001). The mice were 8 weeks old and weighed 16–18 g. They were housed in an SPF environment at the Natural Products Research Center of Guizhou Province, with a controlled temperature of 25 ± 1 °C and a 12-h light-dark cycle. Bedding, drinking water, and food were sterilized, and the mice had free access to food and water. Male C57BL/6J mice were castrated after sexual maturity and injected daily with 5 mg/kg testosterone propionate and 0.05 mg/kg phenylestradiol (GLPBIO, Monterey Park, CA, USA) to establish the BPH model. After the operation, the mice were randomly divided into the negative control group, the positive control group, and the treatment group (high and low doses). Eight mice were used in each group. The sample size was determined by power analysis (based on the primary endpoint data from the pilot study, α = 0.05, 1 − β = 0.8). While maintaining exogenous hormone therapy, compound kzl054 was intraperitoneally injected at doses of 30 mg/kg and 60 mg/kg every other day for 28 consecutive days. Measure your weight during the treatment period. After the treatment, blood was collected from the orbital hemorrhage. The serum was centrifuged (at 4000 rpm, 4 °C for 10 min) and stored at −80 °C. The prostate and other organs were quickly removed, weighed, and stored at −80 °C or fixed in fixative solution (Solarbio, China) for histopathology.

### 2.7. Hematoxylin and Eosin (H&E) Staining

Fixative solution-fixed prostate tissue was embedded in paraffin and sectioned with a paraffin sectioning machine. According to the standard procedure, 4 μm sections were taken for H&E staining (Solarbio, China) and histopathological examination.

### 2.8. Immunohistochemistry (IHC) Staining

For IHC staining, prostate tissue sections were incubated overnight at 4 °C with the addition of primary antibodies (HUBIO, Hsinchu City, Taiwan) after antigen repair. Then, treat with the corresponding secondary antibody (HUBIO, China) at room temperature for 1 h. Finally, the sections were stained with diaminobenzidine (Solarbio, China) and hematoxylin reverse staining to observe the positive signal. Images were captured using a microscope (Leica, Wetzlar, Germany) and analyzed using Image-Pro Plus 6.0 software.

### 2.9. Immunofluorescence (IF) Staining

BPH-1 and WPMY-1 cells were inoculated into the upper and lower chambers of the Transwell system, respectively. After co-culture treatment, the cells were washed twice with pre-cooled PBS, fixed in 4% PFA for 20 min, and then washed again with PBS. Then treat the cells with the permeate, wash them three times with PBS, and seal them for 30 min. Add the primary antibody (1:1000 dilution, HUBIO, China) and incubate overnight in a wet room at 4 °C. After recovering the primary antibody, add the secondary antibody (HUBIO, China), incubate at room temperature for 50 min, and wash with PBS. Finally, DAPI nuclear staining (Solarbio, China) was performed, the upper lumen membrane was cut off, the cover plate was removed, and confocal microscopy (Leica, Germany) imaging was carried out. Positive staining was analyzed using Image-Pro Plus 6.0 software.

### 2.10. Western Blotting

Total protein was extracted from prostate tissue using RIPA lysis buffer (Beyotime, Shanghai, China) containing 1% PMSF (Solarbio, China) and protease inhibitor (approximately 450 μL). The protein concentration was determined using the BCA protein assay kit (Beyotime, China). Equal amounts of samples were loaded into a 10% SDS-PAGE gel and transferred onto PVDF membranes (Millipore, Bedford, MA, USA). After blocking with 5% skimmed milk, incubate overnight with primary antibodies (HUBIO, China) at 4 °C, and then incubate with the corresponding secondary antibodies (HUBIO, China) at room temperature for 1 h. Visualize the bands using ECL detection reagents (Beyotime, China) and capture the images using a chemiluminescence instrument (BioRad, Hercules, CA, USA). The band intensity was quantified by ImageJ software (version 1.54g, National Institutes of Health, Bethesda, MD, USA), and anti-GAPDH antibody was used as the sample loading control.

### 2.11. ELISA Detection

Prepare the working solution according to the ELISA kit instructions (Beyotime, China) and collect the supernatant of the treated cell culture medium. The absorbance was measured at 570 nm and 450 nm, and the results were analyzed using a microplate reader. TGF-β1 samples require activation treatment, while other growth factors do not.

### 2.12. Target Prediction

The SMILES notation corresponding to the active compound kzl054 was uploaded to the Swiss Target Prediction (http://www.swisstargetprediction.ch/, accessed on 19 November 2025) and TargetNet (http://targetnet.scbdd.com/calcnet/index/, accessed on 19 November 2025) databases to screen for potential targets. The predicted targets were compiled and corrected to standard gene names based on the human species using the UniProt database (https://www.uniprot.org/, accessed on 19 November 2025). A list of target proteins was obtained from the GeneCards database (https://www.genecards.org/, accessed on 19 November 2025), and a Venn diagram of the target proteins was created using Venny 2.1.0 (https://bioinfogp.cnb.csic.es/tools/venny/index.html, accessed on 19 November 2025).

### 2.13. Molecular Docking

The 2D structure of kzl054 was drawn by using ChemBioDraw 14, and the 2D structure was converted to 3D and minimized using ChemBio3D Ultra 14.0.0.117. The docking target entry number was obtained from UniProt, and the 3D structure of the target was downloaded from the PDB database. Water molecules and ligands were removed using PyMOL 2.5.0. Hydrogen atoms were added using AutoDockTools 1.5.7, and active pockets were identified. Molecular docking was performed using AutoDock Vina 1.2.0 with a maximum energy difference set to 5. Visualization analysis was conducted using PyMOL and Discovery Studio 4.5.

### 2.14. Microtubule Binding Site Assay

In the experiment, tubulin was redissolved in tubulin buffer (80 mM PIPES, pH 6.9, 0.5 mM EGTA, 2.0 mM MgCl_2_) to a final concentration of 1 mg/mL. Paclitaxel and colchicine were dissolved in DMSO to final concentrations of 2 μmol/L and 3 μmol/L, respectively. On a 96-well plate, 5 μL of tubulin (CST, Danvers, MA, USA), paclitaxel, and colicine (GLPBIO, USA) were added to the Wells of the control group, while 5 μL of a compound with a concentration of 10 μmol/L was added to the wells of the experimental group, and the process was repeated. Incubate at 37 °C and read the absorbance at 340 nm per minute for kinetic analysis by using a microplate reader (Gene, Hong Kong).

### 2.15. EBI Competitive Binding Assay

BPH-1 cells were seeded on 6-well plates at a density of 5 × 10 cells per well. After 24 h, different concentrations of compounds (0.2 μmol/L, 1 μmol/L, 5 μmol/L, 25 μmol/L) were added, respectively, as the experimental groups, colchicine (5 μmol/L) and chimonine (5 μmol/L, 25 μmol/L) as the control groups, and a blank control group was also set up. After treatment for 2 h, add 100 μmol/L N,N′-ethylenedibis (iodoacetamide) (EBI) (Beyotime, China) for another 1.5 h. Collect cells and lyse them to obtain proteins. Proteins were isolated using SDS-PAGE (10% gel, 90 V, 30 min, 120 V, 1.5 h) and transferred onto membranes for Western blot analysis.

### 2.16. Data Processing and Analysis

All data are expressed as mean ± SD. Data collation, analysis and plotting were performed using GraphPad Prism 7.00 software. Differences between groups were analyzed by ANOVA. * *p* < 0.05, ** *p* < 0.01.

## 3. Results

### 3.1. kzl054 Inhibits the Cell Growth of BPH-1 and WPMY-1 Cells In Vitro

**Figure 1:**
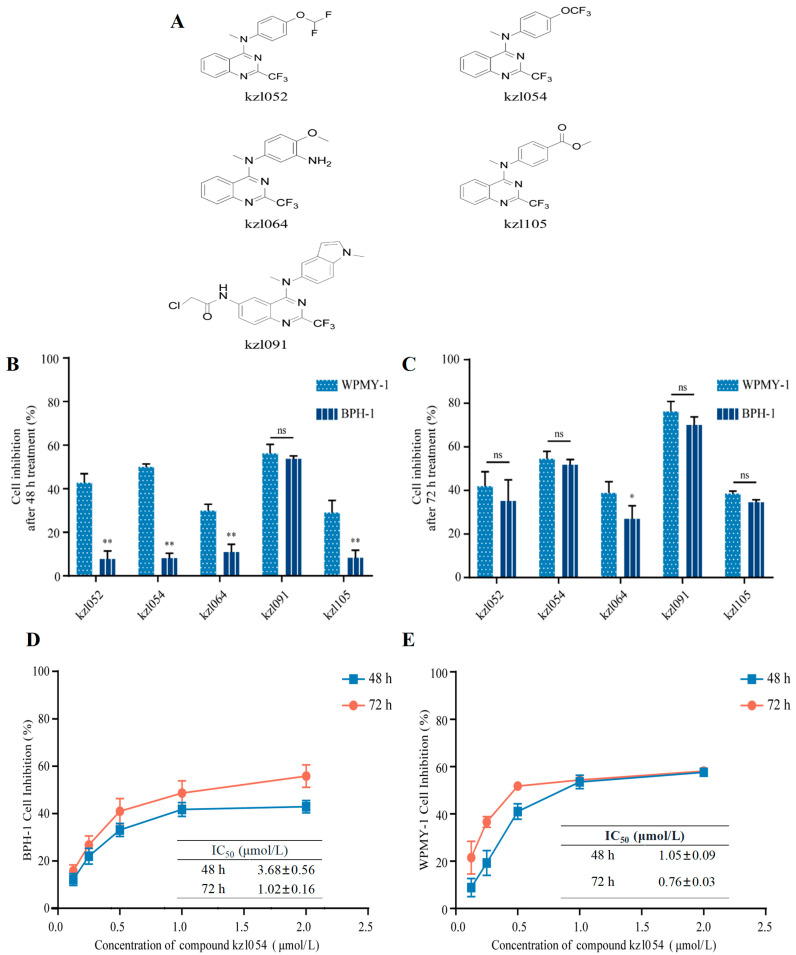
Activity screening of trifluoromethyl quinazoline derivatives. (**A**) Chemical structure of trifluoromethyl quinazoline derivatives; Growth inhibition on BPH-1 and WPMY-1 cells by trifluoromethyl quinazoline derivatives at a concentration of 1.0 μmol/L for 48 h (**B**) and 72 h (**C**), respectively. Dose–response curves of growth inhibition on BPH-1 and WPMY-1 cells by kzl054 after 48 h (**D**) and 72 h (**E**) of action, respectively. Dose–response curves of growth inhibition on BPH-1 and WPMY-1 cells by kzl091 after 48 h and 72 h of action, respectively. The control group was DMSO, and the results were expressed as mean ± SD. The difference results are expressed as * *p* < 0.05, ** *p* < 0.01.

Five compounds (Figure 1A) with inhibitory activity against the growth of BPH-1 cells were screened from a series of tricin derivatives synthesized by our research group in the previous stage. The inhibitory activities of the five compounds (1.0 μmol/L) against the growth of BPH-1 and WPMY-1 cells were detected by the MTT method at 48 h (Figure 1B) and 72 h (Figure 1C), respectively. The results showed that only kzl091 had significant inhibitory activity against the growth of both cell types after 48 h of treatment. After 72 h treatment, kzl054 and kzl091 had inhibitory activities of more than 50% against WPMY-1 and BPH-1 cells, among which kzl091 had a stronger inhibitory activity, with inhibition rates of more than 70% for both cell types at a concentration of 1.0 μmol/L. Further cytotoxicity evaluation was conducted using normal liver cells and liver cancer cells. The SI values of kzl054 at 48 h and 72 h were 10.9 and 9.72, respectively, which were significantly (*p* < 0.01) higher than those of kzl091. In vitro at the cellular level, kzl054 demonstrated significantly higher IC50 values against normal hepatocytes (L-02) than against BPH-1 cells (or your BPH-related cell lines), indicating a higher calculated selectivity index. This preliminary finding suggests that kzl054 may carry lower hepatotoxicity risks at concentrations exhibiting proliferative inhibitory effects, providing support for further in vivo safety evaluation. Therefore, kzl054 was selected for in-depth exploration of its inhibitory activity and mechanism of action on the growth of prostate hyperplasia cells and their stromal cells. The results showed that kzl054 had significant dose- and time-dependent inhibitory activity against the growth of BPH-1 and WPMY-1 cells at 48 h (Figure 1D) and 72 h (Figure 1E). The IC50 values of kzl054 for the two cell types after 48 h treatment were 3.68 ± 0.56 and 1.05 ± 0.09 μmol/L, respectively, and after 72 h treatment, they were 1.02 ± 0.16 and 0.76 ± 0.03 μmol/L, respectively. The results indicated that the inhibitory activity of kzl054 against the growth of WPMY-1 cells was significantly (*p* < 0.01) stronger than that against BPH-1 cells (Table 1).

### 3.2. kzl054 Induces Cell Cycle Arrest and Inhibits Colony Formation in BPH-1 and WPMY-1 Cells

**Figure 2:**
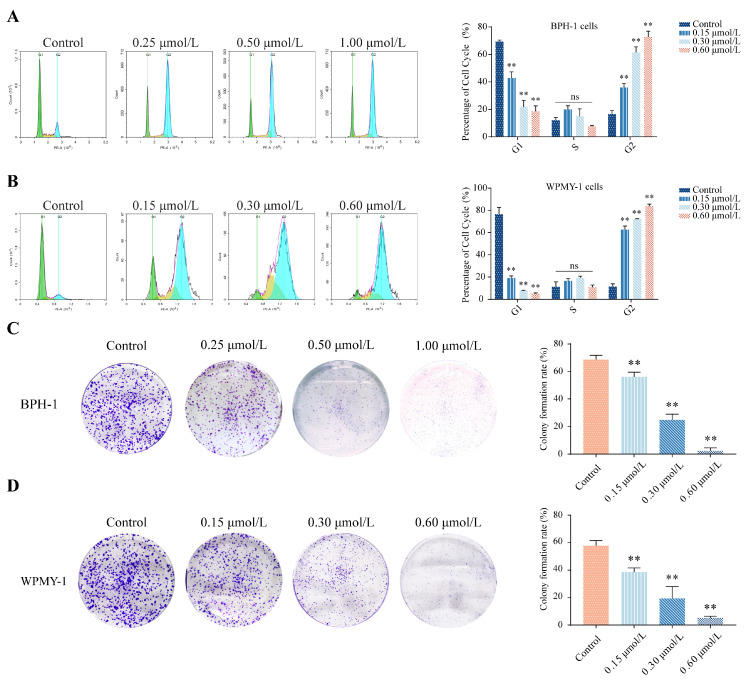
kzl054 inhibits cell cycle arrest and colony formation in BPH-1 and WPMY-1 cells. The cell cycles of BPH-1 (**A**) and WPMY-1 (**B**) cells and their statistical results after kzl054 treatment for 48 h. The colony formation of BPH-1 (**C**) and WPMY-1 (**D**) cells and their statistical results after kzl054 treatment. All data were expressed as mean ± SD, ** *p* < 0.01.

The effects of different concentrations of kzl054 (0.25 μmol/L, 0.50 μmol/L, 1.00 μmol/L) on the cell cycle and proliferation of WPMY-1 and BPH-1 cells were detected by flow cytometry. Compared with the control group, kz1054 treatment for 48 h led to cell cycle arrest in both cell types, characterized by a reduction in G1 phase cells and an increase in G2 phase cells. The proportion of G2 phase cell arrest increased with the increase in concentration (Figure 2A,B). The colony formation experiment showed that kzl054 significantly inhibited the colony formation ability of the two types of cells in a dose-dependent manner. The colony formation rates of BPH-1 and WPMY-1 cells decreased significantly with the increase of concentration (*p* < 0.01) (Figure 2C,D). These results indicate that kzl054 significantly inhibits the G2/M phase cell cycle and colony formation of BPH-1 and WPMY-1 cells, with a certain concentration dependence.

### 3.3. kzl054 Suppresses Prostate Hyperplasia in C57BL/6J Mice

**Figure 3:**
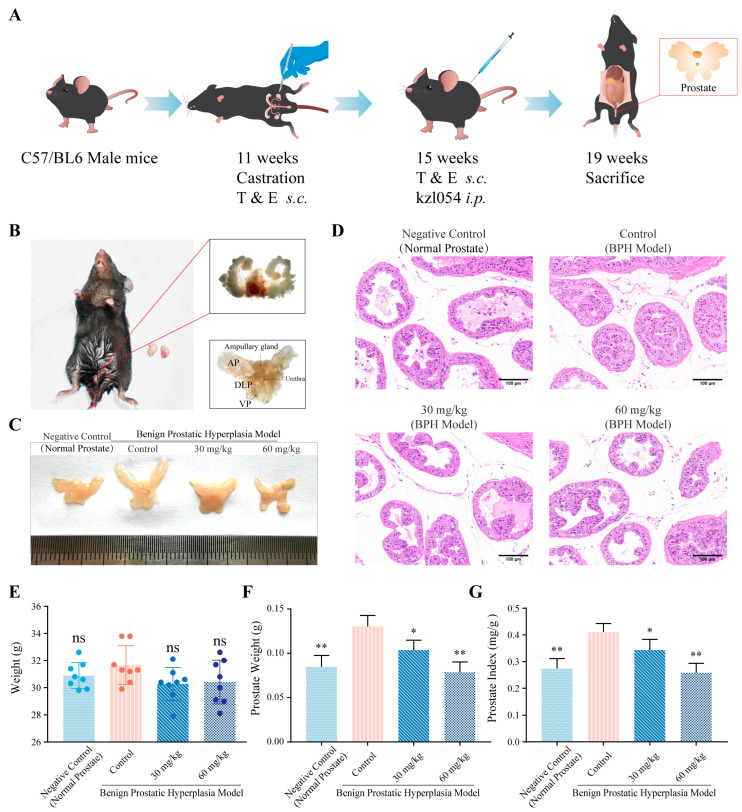
kzl054 inhibits Benign Prostatic Hyperplasia in vivo. (**A**) Model establishment and treatment protocol: After 4 weeks of castration and hormonal stimulation, the mice were treated with kzl054 at doses of 30 mg/kg and 60 mg/kg for 4 weeks, and then the prostate tissue was taken for analysis. (**B**) Schematic diagram of mouse castration surgery, showing the anatomical structure of the prostate. (**C**) The prostate volume of mice in each group after 4 weeks of kzl054 treatment. (**D**) HE staining results of prostate tissues in each group of mice. (**E**) Body weight of mice in each group after treatment. (**F**) Prostate weight of mice in each group. (**G**) Prostate index (PI) of mice in each group. All data were the mean ± SD, * *p* < 0.05, ** *p* < 0.01.

To evaluate the in vivo inhibitory effect of kzl054 on BPH, we established a BPH model by castrating C57BL/6J mice and then stimulating them with testosterone propionate and phenylmethyl estradiol (Figure 3A,B). Compared with the normal group, the prostate tissue of mice in the model group was significantly enlarged, while the high-dose (60 mg/kg) and low-dose (30 mg/kg) kzl054 treatment groups were comparable to the normal group (Figure 3C). The H&E staining results showed that compared with the normal group, the prostate epithelial cells in the model group proliferated significantly, the cell layer thickened and elevated, and papillary protrusions entered the lumen. After kzl054 treatment, the proliferation of prostate epithelial cells was significantly reduced, the cell layer became thinner, and the inwardly protruding epithelial structure decreased (Figure 3D). There was no significant difference in body weight between the model group and the normal group (Figure 3E), indicating that exogenous testosterone and estradiol had no effect on the growth of mice. The prostate weight of mice in the model group was significantly higher than that in the normal group and the kzl054 group, and the prostate index (PI) was significantly increased (Figure 3F). However, the prostate index in the kzl054 group was significantly lower than that in the model group, showing a dose-dependent relationship (*p* < 0.05; *p* < 0.01) (Figure 3G). These results indicate that the combined stimulation of exogenous testosterone and estradiol can induce BPH in castrated male mice, while kzl054 can significantly inhibit this process.

**Figure 4:**
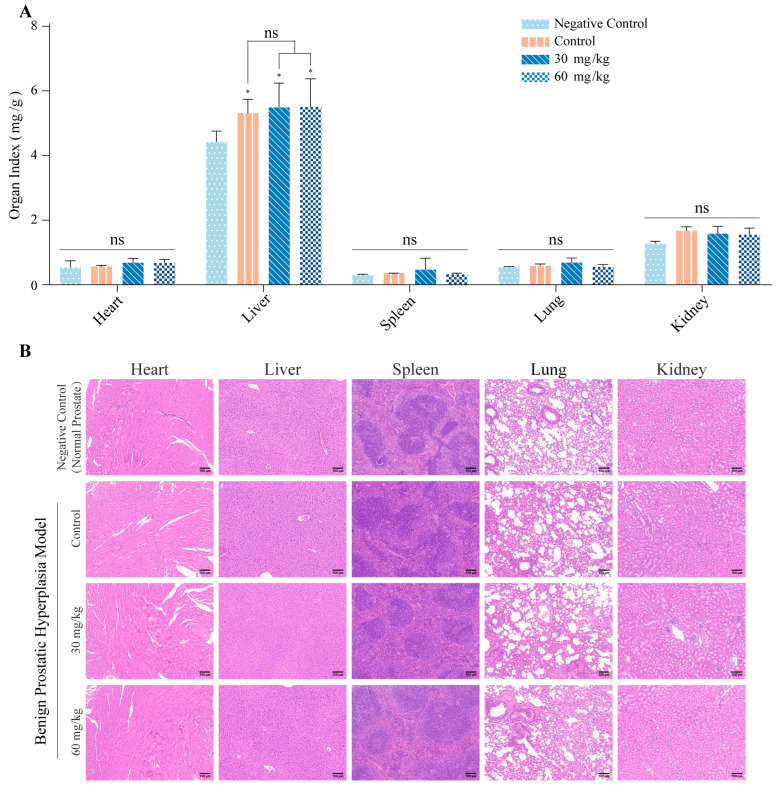
Major organ index and H&E staining results among each group. (**A**) Organ index of the heart, liver, spleen, lungs and kidneys in each group of mice. (**B**) H&E staining results of the heart, liver, spleen, lung and kidney of mice in each group. Scale = 100 μm. All results were expressed as mean ± SD, with * *p* < 0.05.

In addition, there was no statistical significance in the body weight, heart, spleen and lung weight of mice in each group after treatment. However, there were statistically significant differences in the liver between the normal group and the model group, as well as the kzl054 group (*p* < 0.05), while there was no statistically significant difference between the kzl054 group and the model group (Figure 4A). Meanwhile, no abnormalities were observed in the H&E staining of the main organs of mice in each group (Figure 4B). The above results suggest that kzl054 has no significant effect on the organs of mice.

### 3.4. kzl054 Inhibits TGF-β1 Secretion in Co-Cultured WPMY-1 Cells

**Figure 5:**
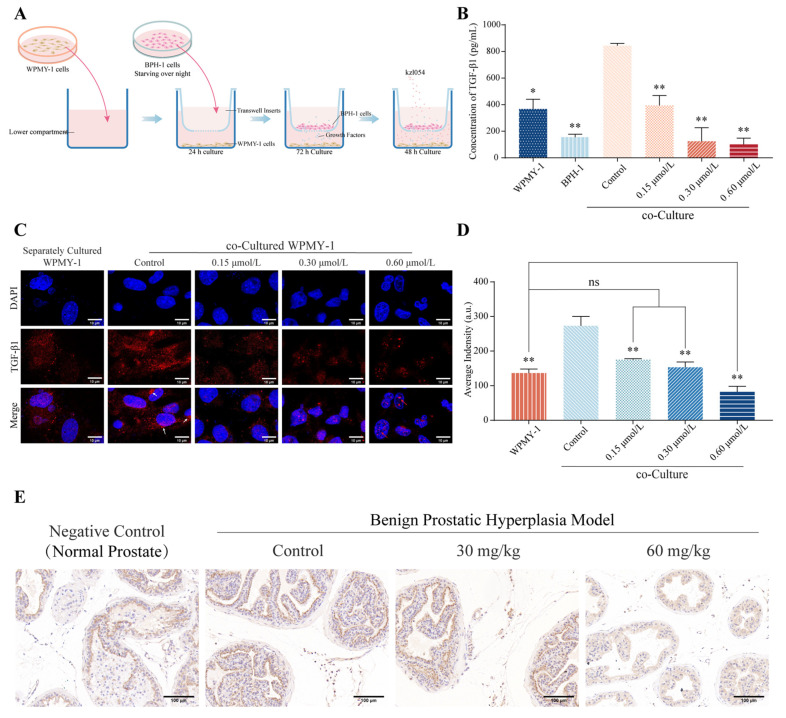
kzl054 inhibits the secretion of TGF-β1 in co-cultured WPMY-1 cells. (**A**) Model diagram of the co-culture system of BPH-1 and WPMY-1 cells. (**B**) The concentration of TGF-β1 in each group of the co-culture system. (**C**) Immunofluorescence staining results of TGF-β1 in WPMY-1 cells. White arrow: TGF-β1 in the cytoplasm. Red arrow: TGF-β1 in the nucleus. (**D**) Semi-quantitative analysis of fluorescence intensity. Scale = 10 μm. (**E**) Immunohistochemical results of TGF-β1 in the prostate tissue of mice. Scale = 100 μm. All data were the mean ± SD, * *p* < 0.05, ** *p* < 0.01.

To analyze the effect of kzl054 on the secretion of inflammatory factors by WPMY-1 cells, we established a co-culture system of prostate stromal WPMY-1 cells and BPH-1 epithelial cells (Figure 5A). Different concentrations of kzl054 were added to the co-culture system for treatment to evaluate the secretion of growth factors TGF-β1, IGF-1, EGF and VEGF. ELISA results showed that the level of TGF-β1 in the co-culture group was significantly higher than that in the monoculture group (*p* < 0.01; *p* < 0.05) (Table 2). However, kzl054 treatment significantly reduced the secretion of TGF-β1 (*p* < 0.01) (Figure 5B). Immunofluorescence staining showed that when WPMY-1 cells were co-cultured with BPH-1 cells, the expression of TGF-β1 could be enhanced, while kzl054 treatment could reduce the extracellular distribution of TGF-β1 (Figure 5C). Semi-quantitative fluorescence intensity analysis further confirmed that kzl054 treatment significantly reduced the expression of TGF-β1 (Figure 5D). The immunohistochemical staining results of the prostate tissue of mice showed that TGF-β1 was strongly positively expressed in the model group, while TGF-β1 was significantly weakened after treatment with 60 mg/kg kzl054 (Figure 5E). These results suggest that kz1054 may inhibit the progression of BPH by suppressing the secretion of TGF-β1 by WPMY-1 cells.

**Table 1:** Selective Index of compound kzl091 in vitro. ** denotes *p* < 0.01.

Compounds	IC_50_/L-02 (μmol/L)		IC_50_/HepG2 (μmol/L)		Selective Index (SI)	
	48 h	72 h	48 h	72 h	48 h	72 h
kzl054	7.50 ± 0.18	3.50 ± 0.13	0.69 ± 0.27	0.36 ± 0.05	10.9 **	9.72 **
kzl091	4.93 ± 0.48	3.33 ± 0.09	1.02 ± 0.11	0.66 ± 0.01	4.83	5.04

**Table 2:** Effect of kzl054 on the level of growth factors in co-culture system. * *p* < 0.05 and ** *p* < 0.01 were considered to indicate statistically significant differences.

Groups	Growth Factors (pg/mL)			
	IGF-1	TGF-β1	EGF	VEGF
BPH-1	NA	155.13 ± 18.35 **	NA	NA
WPMY-1	NA	367.53 ± 59.27 *	NA	NA
Co-cultured control	NA	743.63 ± 14.10	NA	NA
Co-culture (0.15 μmol/L)	NA	394.17 ± 61.01 **	NA	NA
Co-culture (0.30 μmol/L)	NA	124.53 ± 83.88 **	NA	NA
Co-culture (0.60 μmol/L)	NA	101.00 ± 38.99 **	NA	NA

### 3.5. kzl054 Inhibits EMT in Co-Cultured BPH-1 Cells

**Figure 6:**
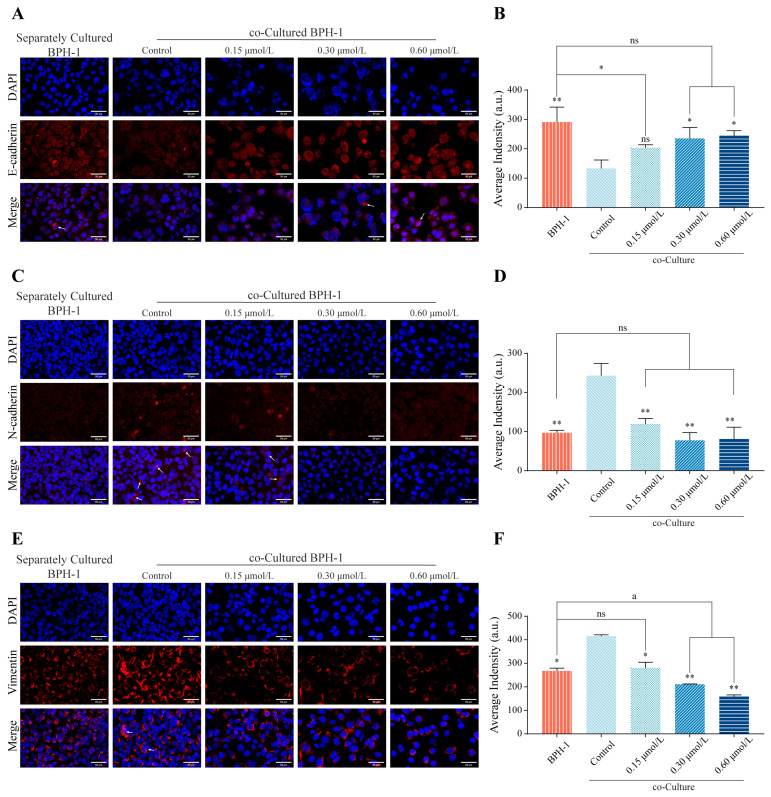
kzl054 inhibits the EMT of BPH-1 cells. (**A**) Immunofluorescence staining results of E-cadherin. White arrow: E-cadherin. (**B**) Semi-quantitative analysis of fluorescence intensity. (**C**) Immunofluorescence staining results of Vimentin. White arrow: Vimentin. (**D**) Semi-quantitative analysis of fluorescence intensity. (**E**) Immunofluorescence staining results of N-cadherin in each group of BPH-1 cells. White arrow: N-cadherin. (**F**) Semi-quantitative analysis results of fluorescence intensity. Scale = 50 μM. All data were the mean ± SD, * *p* < 0.05, ** *p* < 0.01. Compared with the BPH-1 monoculture group, ^a^ *p* < 0.05.

To further analyze the effect of kzl054 on EMT, we used immunofluorescence staining to detect the expression of key markers of the EMT process in co-cultured BPH-1 cells. The results showed that the epithelial marker E-cadherin was downregulated in co-cultured BPH-1 cells, but significantly up-regulated after treatment with kz1054 (Figure 6A,B). For the mesenchymal marker Vimentin, BPH-1 cells cultured alone showed that Vimentin was located near the nucleus, while co-culture led to enhanced expression of Vimentin and changes in cell morphology. After treatment with kzl054, the expression of Vimentin in BPH-1 cells decreased, the number of cells decreased, protein stretching weakened, and the fluorescence intensity also significantly decreased (Figure 6C,D). In addition, the expression of N-cadherin was weak in BPH-1 cells cultured alone, while co-culture with WPMY-1 cells significantly enhanced the expression of N-cadherin in BPH-1 cells, and the addition of kzl054 treatment significantly reduced the expression of N-cadherin (Figure 6E,F). These results indicate that kzl054 significantly inhibits the EMT process of co-cultured BPH-1 cells.

### 3.6. kzl054 Inhibited Microtubule Polymerization by Targeting β-Tubulin

**Figure 7:**
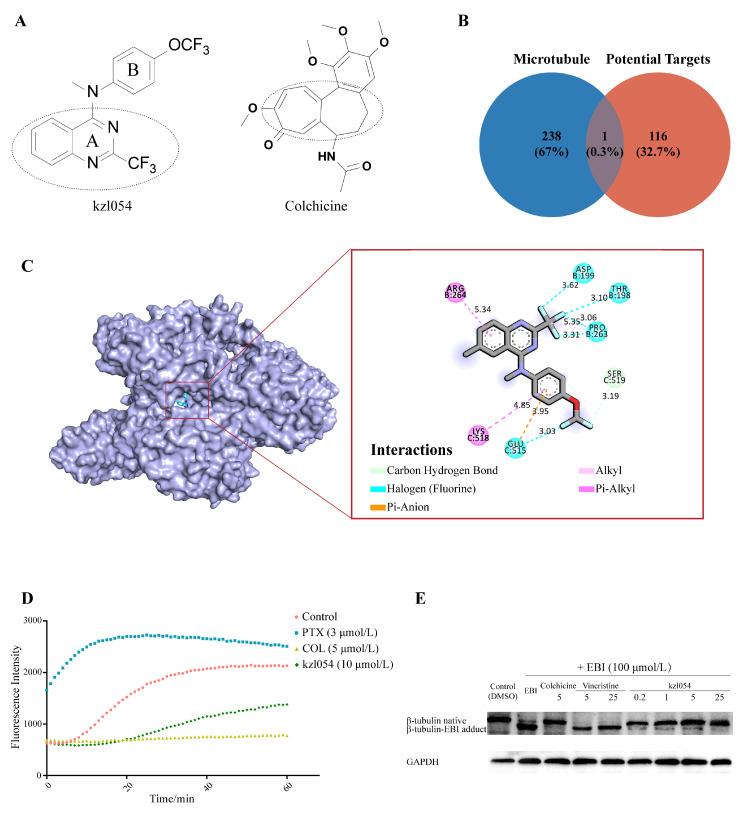
kzl054 competitively binds to β-tubulin and inhibits the level of TGF-β1. (**A**) Structural comparison between kzl054 and microtubule inhibitor Colchicine. (**B**) Potential targets of microtubule-associated proteins and active compound kzl054 were analyzed by Venny. (**C**) Visualization results of kzl054 binding to β-tubulin protein (β-tubulin PDB: 1TUB). (**D**) 10 μmol/L kzl054 inhibited microtubule polymerization, compared with paclitaxel (promoting polymerization) and colchicine (inhibiting polymerization) controls. (**E**) EBI competitive assay showed that kzl054 could bind colchicine site and reduce the binding of EBI to β-tubulin.

To further explore the molecular mechanism of kzl054 on BPH, it was found through structural comparison that kzl054 has a similar core structure to colchicine (Figure 7A). Through database prediction and screening, it was found that β-tubulin is a potential target for kzl054 in the treatment of BPH (Figure 7B). The molecular docking results indicated that the binding energy of kzl054 to β-tubulin was −8.0 kcal/mol, which was higher than that of colchicine (−7.9 kcal/mol) (Figure 7C). The microtubule polymerization experiment confirmed that kzl054 inhibited the polymerization of microtubulin. Its fluorescence intensity within 30 min was lower than that of the paclitaxel group and the control group, and was similar to that of the colchicine positive control group (Figure 7D). Further EBI competitive inhibition experiments demonstrated that kzl054 could bind to the colchicine binding site on β-tubulin, preventing its specific modification and inhibiting microtubule polymerization (Figure 7E). These results suggest that kz1054 may alleviate the progression of BPH by targeting β-tubulin and inhibiting microtubule polymerization.

### 3.7. kzl054 Inhibits β-Tubulin Expression and Localization in Co-Cultured WPMY-1 and BPH-1 Cells

**Figure 8:**
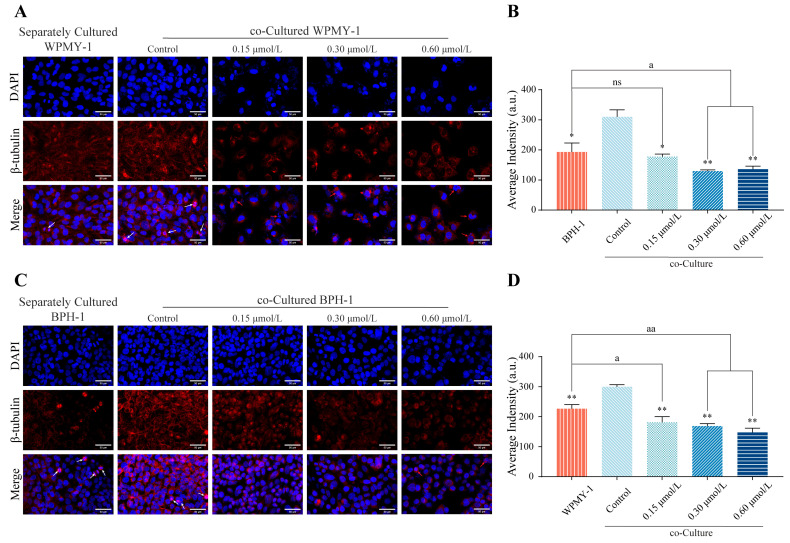
Effect of kzl054 on β-tubulin in WPMY-1 and BPH-1 co-cultured cells. (**A**) Immunofluorescence staining of WPMY-1 cells. (**B**) Semi-quantitative analysis of fluorescence intensity. (**C**) Immunofluorescence staining of BPH-1 cells. (**D**) Semi-quantitative analysis of fluorescence intensity. Scale = 50 μm, White arrows: Mitotic cells with high expression of β-tubulin, Red arrows: Apoptotic cells. Compared with the co-culture control group, * *p* < 0.05 and ** *p* < 0.01; compared with the BPH-1 monoculture group, ^a^ *p* < 0.05 and ^aa^ *p* < 0.01.

In addition, we also analyzed the effect of kzl054 on the expression and localization of β-tubulin in WPMY-1 and BPH-1 co-cultured cells. After co-culture for 72 h, different concentrations of kzl054 (0.15 μmol/L, 0.30 μmol/L, 0.60 μmol/L) were added and co-cultured for 48 h. The expression and localization of β-tubulin in the two types of cells were detected by immunofluorescence. The results showed that kzl054 treatment led to a significant reduction in β-tubulin expression and disrupted localization in WPMY-1 and BPH-1 cells (Figure 8). These findings suggest that kzl054 may inhibit the progression of BPH by targeting and suppressing the expression and localization of β-tubulin.

## 4. Discussion

In this study, kzl054 demonstrated significant anti-proliferative activity against both prostate epithelial cells and stromal cells, effectively inducing apoptosis and inhibiting cell proliferation. The in vivo efficacy and safety of kzl054 in improving BPH were further verified in the established mouse BPH model. Unlike other studies [33,34], we first performed castration surgery on model mice to avoid interference from the self-secretion of androgens, and successfully established a BPH model by continuously administering testosterone and estradiol. Treatment with different doses of kzl054 can significantly reduce the prostate index and effectively inhibit prostatic hyperplasia.

It is important to note that the selective index calculated herein, based on hepatocyte toxicity, is an initial step in safety profiling. While a high SI suggests a reduced risk of hepatotoxicity at efficacious doses, it does not preclude potential effects on other organs. A more comprehensive evaluation of tissue selectivity would require future studies comparing the compound’s effects on primary benign prostatic hyperplasia cells versus normal prostatic epithelial and stromal cells. Nevertheless, the favorable SI observed supports the rationale for proceeding to in vivo efficacy and safety studies in relevant animal models.”

The interaction between prostate epithelial cells and stromal cells can trigger the transition and migration of epithelial cells to mesenchymal cells, promoting excessive proliferation of the prostate. Growth factors secreted by stromal cells (such as TGF-β1) can induce EMT in epithelial cells, and inhibiting TGF-β1 can reverse this process [35]. TGF-β1 not only enhances the invasiveness of cells by inducing angiogenesis, inhibiting apoptosis and increasing cell motility, but also promotes BPH through an unbalanced proliferation-apoptosis process [36]. When BPH-1 cells were cultured in the medium containing TGF-β1, their in vitro healing ability was enhanced, suggesting that TGF-β1 might promote proliferation by enhancing the migration ability of BPH-1 cells to transform into interstitial cells [37]. Therefore, targeting the interaction between BPH-1 and WPMY-1 cells is a promising strategy for improving BPH.

Positioning KZL054’s Mechanism within the Current BPH Therapeutic Landscape. The findings that kzl054 modulates β-tubulin and downstream pathways invite a discussion on its differentiation from existing therapies. Unlike α1-blockers, which only relieve symptoms, or 5α-reductase inhibitors, which primarily target the epithelial compartment, kzl054’s potential action on the cytoskeleton directly addresses the aberrant stromal proliferation and fibrosis that contribute to the ‘static’ component of bladder outlet obstruction. This mechanism is conceptually distinct and may complement current approaches. However, it is crucial to acknowledge that kzl054 is part of a known chemical class. Its potential novelty lies in its specific application to BPH pathology and its observed preliminary safety window in vitro, which distinguishes it from cytotoxic chemotherapeutic microtubule inhibitors. Future studies comparing kzl054 head-to-head with standard BPH drugs and other microtubule agents will be essential to define its relative advantage.

Compared with other studies, this study established an in vitro co-culture system of BPH-1 and WPMY-1 cells to simulate the matrix microenvironment of BPH-1 cells. Under the influence of growth factors secreted by stromal cells, such as TGF-β1, BPH-1 cells undergo EMT, which is manifested by the down-regulation of epithelial marker E-cadherin, the up-regulation of mesenchymal markers N-cadherin and Vimentin, and the increase of β-tubulin expression. kzl054 can not only significantly inhibit the proliferation of WPMY-1 cells, but also reduce the expression and secretion of TGF-β1 in WPMY-1 cells, thereby inhibiting the EMT of BPH-1 cells induced by TGF-β1. Further mechanism studies have shown that kzl054 binds to β-tubulin, competitively inhibits the binding of colicine, inhibits microtubule aggregation, down-regulates the expression and secretion of TGF-β1, and ultimately inhibits the proliferation of BPH-1 and WPMY-1 cells. Although this study clarified the potential mechanism by which kzl054 alleviates BPH. However, the specific mechanism by which kzl054 targets β-tubulin to inhibit TGF-β1 and its signaling pathway remains unclear.

While the present study demonstrates the efficacy of KZL054 in a preclinical BPH model, its potential clinical relevance must be considered within the landscape of existing standard therapies, primarily 5-α-reductase inhibitors (e.g., finasteride) and α1-adrenergic receptor blockers (e.g., tamsulosin) [38]. Finasteride reduces prostate volume by suppressing the androgenic pathway but has a slow onset and is associated with sexual side effects. α-blockers provide rapid symptomatic relief by relaxing smooth muscle but do not alter disease progression [39]. The mechanism of action of KZL054, which appears to involve simultaneous induction of apoptosis in both epithelial and stromal compartments, or specific anti-fibrotic effects, suggests a distinct profile. It may offer the dual potential to reduce prostate volume and alleviate dynamic obstruction, possibly with a different side effect profile. However, a direct head-to-head comparison of KZL054 with these standard drugs in preclinical models is necessary to quantitatively validate any potential superiority or complementary effect and is a critical next step in its translational development. This study lays the mechanistic foundation for such future comparative evaluations.

## 5. Conclusions

Fluoroquinazoline derivative kzl054 inhibits the progression of BPH by targeting β-tubulin to reduce TGF-β1 secretion in WPMY-1 cells and inhibits EMT in BPH-1 cells. This study provides new theoretical mechanisms and therapeutic strategies for the treatment of refractory BPH.

## Data Availability

The original contributions presented in this study are included in the article. Further inquiries can be directed to the corresponding authors.
